# Effects of commercial genetic selection on gene expression in the developing neuroendocrine system of broilers

**DOI:** 10.1016/j.psj.2025.106331

**Published:** 2025-12-23

**Authors:** Panpradub Sinpru, Kristen Diehl, Laura E. Ellestad, Tom E. Porter

**Affiliations:** a Department of Animal and Avian Sciences, University of Maryland, College Park, MD, 20742, USA; b Animal Biosciences and Biotechnology Laboratory, BARC, ARS, USDA, Beltsville, MD, 20705, USA; c Department of Poultry Science, University of Georgia, Athens, GA 30602, USA

**Keywords:** Broiler, Embryonic development, Genetic selection, Gene expression, Neuroendocrine system

## Abstract

Selective breeding of broilers has significantly improved growth rates, muscle mass, and feed efficiency and may have influenced the neuroendocrine systems that regulate growth and metabolism. Embryonic development represents one-third of the life of a modern broiler. To assess the impact of genetic selection on the neuroendocrine regulation of growth and metabolism during embryonic development, we examined mRNA expression of growth-related genes in the hypothalamus and anterior pituitary of two chicken breeds: the modern Ross 708 broiler and the Athens Canadian Random Bred (ACRB) line, the oldest established strain for meat-type chickens. Hypothalami and pituitary glands were dissected from embryos at days 10, 12, 14, 16, and 18 of incubation (*n* = 4 for each combination of breed, age, and gender). Levels of mRNA for each target gene were quantified using reverse transcription-quantitative PCR. In the adrenocorticotropic axis, pituitary corticotropin-releasing hormone receptor 1 mRNA levels were influenced by the interaction of breed, age, and gender (*P* < 0.05). In the thyrotropic axis, pituitary thyroid-stimulating hormone β-subunit mRNA levels were affected by the interaction of breed, age, and gender (*P* < 0.05). In the somatotropic axis, mRNA levels of hypothalamic somatostatin were higher in ACRB than Ross, whereas overall pituitary growth hormone mRNA levels were greater in Ross than ACRB (*P* < 0.05). Pituitary growth hormone-releasing hormone receptor 2 and pituitary adenylate cyclase-activating polypeptide receptor 1 were influenced by the interaction between breed and age (*P* < 0.05). In the gonadotropic axis, hypothalamic gonadotropin-releasing hormone 1 and gonadotropin-inhibiting hormone were influenced by breed, age, and gender, while mRNA levels of pituitary follicle-stimulating hormone β-subunit were affected by the interaction between breed and age (*P* < 0.05). Hypothalamic agouti-related peptide, neuropeptide Y, and proopiomelanocortin mRNA levels were higher in ACRB than Ross in females (*P* < 0.05). These findings indicate that genetic selection of broilers has altered the adrenocorticotropic, somatotropic, thyrotropic, and gonadotropic axes, as well as hypothalamic control of appetite and metabolism during embryonic development.

## Introduction

Over decades of breeding, broilers have been selected for rapid growth, increased muscle mass, and improved feed efficiency, establishing broilers as the primary source of poultry meat in the global commercial industry (Tavárez and de los Santos, 2016). While advancements have mainly targeted physical traits, the genetic modifications could also influence other vital systems in the body, including the neuroendocrine system, which plays a key role in regulating growth, the stress response, metabolism, reproduction, and other physiological processes (Vaccaro et al., 2022a,b; Zhou et al., 2025). Understanding the physiological changes that contribute to improvements in growth performance will potentially guide further advancements in broiler production efficiency.

Embryonic development is a critical phase in chickens, accounting for approximately one-third of the growing period to market weight in broilers. This highlights the importance of optimized management and breeding strategies that target this stage (Speier et al., 2012). The neuroendocrine system functions through a complex network of hormones and signaling pathways, primarily governed by the hypothalamus and pituitary gland (Yeung et al., 2006; Bédécarrats et al., 2016; Ellestad et al., 2019; Pritchett et al., 2023; Xu et al., 2023). Five neuroendocrine axes are established during embryonic development, with the adrenocorticotropic axis established first, followed by the thyrotropic axis, then the somatotropic axis. The lactotropic axis is established shortly before hatch, while the gonadotropic axis initiates during embryogenesis but is not fully established until well after hatch (Ellestad et al., 2011). Corticotrophs secrete adrenocorticotropic hormone (**ACTH**), which is generated through the proteolytic cleavage of the pro-opiomelanocortin (**POMC**) precursor. The adrenocortical steroids produced through this axis play crucial roles in the metabolism of macronutrients to support the growth of an organism and for its post-embryonic survival (Jenkins and Porter, 2004). Thyroid-stimulating hormone (**TSH**), a glycoprotein produced by the anterior pituitary, consists of a glycoprotein hormone α-subunit and a hormone-specific β-subunit (**TSH-β**) that is synthesized by thyrotrophs. TSH plays a key role in stimulating the production of thyroid hormones in the thyroid gland, particularly thyroxine and triiodothyronine, which are essential for various physiological processes, including metabolism, growth, development, and thermoregulation in birds (McNabb, 2007). Growth hormone (**GH**) is produced by somatotroph cells in the anterior pituitary, and its secretion is stimulated by growth hormone-releasing hormone (**GHRH**) and inhibited by somatostatin (**SST**) (Steyn et al., 2016). GH plays a crucial role in regulating growth and metabolism in chickens (Porter et al., 1995; Reiprich et al., 1995). The secretion of prolactin (**PRL**) from the anterior pituitary gland in birds is stimulated by vasoactive intestinal peptide (**VIP**) (Proudman and Opel, 1988; Kansaku et al., 1995; Bédécarrats et al., 1999). In the gonadotropic axis, the hypothalamus produces gonadotropin-releasing hormone (**GnRH**) to stimulate its receptors on the pituitary gland to secrete luteinizing hormone (**LH**) and follicle-stimulating hormone (**FSH**) (Bédécarrats et al., 2009). In addition, interactions between gonadotropins and GH in modulating growth performance have been reported (Bahry et al., 2023). The release of gonadotropins and their interactions with leptin, sex steroids, and GH play a key role in regulating juvenile growth and body composition (Rogol et al., 2002). A comprehensive understanding of ontogenetic changes in the neuroendocrine system, alongside the anatomical and functional development of communication systems that regulate hormonal signals, is essential, as these are critical for coordinating key developmental processes during hatching and birth (Bruggeman et al., 2002; De Groef et al., 2008). The hypothalamus integrates signals from the brain, peripheral circulation, and gastrointestinal tract to regulate energy balance. This control is mediated mainly by two neuronal populations in the arcuate nucleus (**ARC**): orexigenic agouti-related peptide/neuropeptide-Y (**AgRP/NPY**) neurons, which promote food intake and reduce energy expenditure, and anorexigenic POMC neurons, which suppress feeding and enhance catabolic processes in both birds and mammals (Boswell et al., 2002; Richards, 2003). The development of this network differs across species, occurring primarily after birth in rodents but largely before birth in sheep and humans (Koutcherov et al., 2002; Grove and Smith, 2003; Mühlhäusler et al., 2004). Although data are limited for chick embryos, some evidence suggests that energy homeostasis may be established before hatching in chickens, consistent with their precocial nature (Furuse, 2002; Huang et al., 2008).

The Athens Canadian Random Bred (**ACRB**) line is the oldest established control strain for meat-type chickens, originating in the mid-1950s (Collins et al., 2016). Due to their slower growth, smaller body size, and a higher feed conversion ratio compared to modern broilers, ACRB birds serve as a valuable baseline for studying the effects of genetic selection (Collins et al., 2014; Vaccaro et al., 2022a). Moreover, recent studies have used the ACRB as a model for identifying the role of genetic selection on physiological traits of the modern broiler in the somatotropic axis (Vaccaro et al., 2022a) and corticotropic and thyrotropic axes (Vaccaro et al., 2022b). However, the impact of genetic selection on development of the neuroendocrine system, a key regulator of various physiological processes in the body, is not well understood. Therefore, this study aimed to examine mRNA expression of growth- and metabolism-related genes in the hypothalamus and anterior pituitary of Ross 708 broiler and ACRB birds. The identified differences may contribute to the enhanced growth traits observed in modern broilers, and the knowledge gained could lead to the development of more targeted breeding approaches that enhance production efficiency while minimizing potential adverse effects on health, welfare, and reproduction.

## Materials and methods

### Animals and tissue collection

Fertilized eggs from a modern commercial line (Ross 708) were obtained from Allen’s Hatchery (Seaford, DE), and fertilized eggs from the ACRB line were supplied by the Department of Poultry Science, University of Georgia. All eggs were transported by the investigators and stored in a cooler maintained at 12 °C until they were simultaneously placed in the incubator. The eggs were incubated at 37.5 °C with 60 % humidity and automatic turning every 3 hours. At embryonic days 10, 12, 14, 16, and 18 (e10, e12, e14, e16, and e18), genders were determined by dissecting embryos under a microscope, with two smooth gonads of roughly equal size indicating males and a single gonad or gonads of greatly different sizes with visible primordial follicles indicating females, as reported by Mittwoch (1998). Then, the hypothalami and anterior pituitary glands were carefully dissected from four male and four female embryos per line on each embryonic day examined. Dissections were performed under a dissecting microscope, following previously published protocols (Porter et al., 1995; Higgins et al., 2010). The collected tissues were immediately frozen in liquid nitrogen and stored at −80 °C until RNA extraction. All procedures were reviewed and approved by the Institutional Animal Care and Use Committee at the University of Maryland.

### RNA extraction and gene expression analysis

Total RNA was extracted from the hypothalami and anterior pituitary glands using RNeasy Mini Kits (Qiagen, Valencia, CA). RNA quantity was determined using the Quant-iT RiboGreen RNA Quantitation Reagent (Invitrogen, Carlsbad, CA). A total of 500 ng of RNA was reverse transcribed into cDNA using SuperScript III (Invitrogen, Carlsbad, CA) and an oligo-dT primer (5′-CGGAATTCTTTTTTTTTTTTTTTTTTTTV-3′). Relative gene expression of key components of the adrenocorticotropic, thyrotropic, somatotropic, lactotropic, and gonadotropic axes in the hypothalamus was quantified by reverse transcription-quantitative PCR (RT-qPCR). Primers and cycling parameters used were described previously by Ellestad et al. (2011) and Higgins et al. (2010). Glyceraldehyde-3-phosphate dehydrogenase (**GAPDH**) was used to normalize target gene expression levels for hypothalamic tissue, and phosphoglycerate kinase 1 (**PGK1**) was used for pituitary tissue. The method of selection of these housekeeping genes was described by Ellestad et al. (2011). Data were normalized to the average expression level of the target gene in male Ross e18 samples and log_2_-transformed prior to statistical analysis. The 2^-ΔΔCT^ method (Livak and Schmittgen, 2001) was used to calculate relative gene expression levels, with four biological replicates, each amplified in duplicate.

### Statistical analysis

Statistical analysis was performed using the mixed model ANOVA procedure in SAS (SAS Institute, Cary, NC). The model included ‘breed’, ‘age’, and ‘gender’ as fixed effects, along with their interactions. A post-hoc test was performed with the PDIFF option and a Tukey adjustment for statistical significance at *P* < 0.05.

## Results

### Pit-1 isoforms

In chickens, three pituitary-specific transcription factor 1 (**Pit-1**) isoforms—Pit-1α, Pit-1β, and Pit-1γ—have been identified (Mukherjee and Porter, 2012). While these isoforms share a conserved C-terminal POU DNA-binding domain, they differ in the N-terminal transactivation domain (Tanaka et al., 1999; Van et al., 2000). Pit-1 is essential for the production of hormones in thyrotrophs, somatotrophs, and lactotrophs (Zhu et al., 2007). We measured the ontogeny of these *Pit-1* isoforms as well as *total Pit-1* transcript levels during embryonic development. The interactions and main effects across breed, age, and gender are shown in Table 1, and the relative mRNA levels of these isoforms are shown in Table 2. No significant interaction or main effects of breed, age, or gender on the mRNA levels of *Pit-1* isoforms or *total Pit-1* were observed during embryonic development (*P* > 0.05).

**Table 1:** Effects of breed, age, and gender on mRNA levels of candidate genes in hypothalamic and pituitary tissues in five neuroendocrine axes. Bold font indicates a statistical significance of the effect (*P* < 0.05).

Gene	Effect						
	Breed	Age	Gender	Breed*Age	Breed*Gender	Age* Gender	Breed*Age* Gender
Adrenocorticotropic axis							
*CRH*	0.6384	**0.0190**	**<0.0001**	0.9006	0.2081	0.2155	0.0710
*CRH-R1*	0.6684	**0.0100**	0.2213	0.2666	0.4867	0.4604	**0.0010**
*CRH-R2*	0.2376	**0.0045**	0.639	0.9244	0.2155	0.5315	0.1528
*POMC*	0.5184	**0.0001**	0.7807	0.9111	0.3566	0.9949	0.6627
Thyrotropic axis							
*TRH*	0.4168	**<0.0001**	0.0951	0.9231	0.7034	0.5322	0.1530
*TRH-R*	0.5394	0.2318	0.1866	0.5327	0.4601	0.9928	0.4794
*TSH-β*	**0.0428**	**<0.0001**	0.3598	0.9273	0.1036	0.3085	**0.0244**
Somatotropic axis							
*GHRH*	0.6551	**0.0001**	**0.0005**	0.4848	0.1670	**0.0009**	0.0595
*PACAP/GHRH-like*	0.5877	**0.0138**	**0.0136**	0.6551	0.9229	0.7029	0.3324
*Ghrelin*	0.3311	0.0716	**<0.0001**	0.4307	0.1315	0.6331	0.3058
*SST*	**0.0197**	0.0793	0.5933	0.9516	0.5009	0.8690	0.9639
*GHRH-R*	**0.0019**	**<0.0001**	0.4156	0.7825	0.5410	0.2287	0.1750
*GHRH-R2*	0.2196	**0.0048**	0.4822	**0.0471**	0.6237	0.2457	0.9128
*PACAP-R1*	**0.0354**	**0.0004**	0.0821	**0.0429**	0.2525	0.4578	0.3470
*GHRH-LPR*	0.5362	0.9915	0.3009	0.8802	0.2409	0.4092	0.5610
*GHS-R*	0.6724	**0.0112**	**0.0329**	0.1553	0.6883	0.9512	0.5236
*SSTR2*	0.1618	0.9420	0.5208	0.8463	0.6503	0.4265	0.8525
*GH*	**0.0022**	**<0.0001**	0.0956	0.0761	0.2381	**0.0299**	0.1761
Lactotropic axis							
*VIP*	0.5572	**<0.0001**	**0.0028**	0.8234	0.8991	0.9674	0.7949
*PRL*	0.2263	**<0.0001**	0.8684	0.9846	0.1338	0.7272	0.4825
Gonadotropic axis							
*GnRH1*	**0.0086**	**<0.0001**	**0.0094**	0.4122	0.0680	0.1860	0.1907
*GnIH*	**0.0187**	**<0.0001**	**0.0001**	0.8262	0.6776	0.7400	0.1578
*FSH*-*β*	0.4036	**0.0131**	**<0.0001**	**0.0225**	0.3175	0.1869	0.1901
*LH*-*β*	0.8701	**0.0056**	**<0.0001**	0.3668	0.0981	0.0907	0.2972
Others							
*Pit-1α*	0.2881	0.1036	0.1566	0.3276	0.4371	0.9711	0.7549
*Pit-1β*	0.9730	0.0908	0.1012	0.1140	0.6413	0.5862	0.3782
*Pit-1γ*	0.9869	0.9242	0.4002	0.7668	0.4794	0.8469	0.9864
*Total Pit-1*	0.9729	0.4470	0.9819	0.3556	0.8652	0.9997	0.9849
Appetite and metabolism							
*NPY*	**0.0441**	**<.0001**	**0.0033**	0.1507	0.1867	0.4153	0.5068
*AgRP*	**0.0160**	**<.0001**	0.8531	0.9719	0.9679	0.0729	0.1606
*POMC*	0.3237	**<.0001**	**<.0001**	0.2784	**0.0350**	0.0792	0.2197

**Table 2:** Relative mRNA levels of the pituitary-specific transcription factor 1 (**Pit-1**) isoforms, *Pit-1α, Pit-1β, Pit-1γ*, and total *Pit-1* during embryonic development. Levels of target mRNA were normalized to levels of *PGK1* mRNA. Results are presented as the means and standard errors of the means after normalization to the mean for male e10 Ross. *P*-values of the effects on target gene mRNA levels are provided in Table 1. There were no significant main or interactive effects of breed, age, or gender on the mRNA levels of *Pit-1* isoforms or total *Pit-1* transcription observed during embryonic development (*P* > 0.05).

Gene Name	Gender	Breed	e10	e12	e14	e16	e18
*Pit-1α*	Male	Ross	0.97 ± 0.28	0.56 ± 0.22	1.87 ± 0.56	1.06 ± 0.14	1.00 ± 0.64
		ACRB	0.89 ± 0.49	1.44 ± 0.65	3.25 ± 2.68	1.37 ± 0.43	0.40 ± 0.20
	Female	Ross	1.02 ± 0.30	1.60 ± 0.83	1.05 ± 0.45	1.93 ± 0.48	0.87 ± 0.22
		ACRB	2.28 ± 1.64	1.83 ± 0.55	1.95 ± 0.51	1.02 ± 0.26	0.36 ± 0.16
*Pit-1β*	Male	Ross	1.11 ± 0.29	0.88 ± 0.15	2.32 ± 0.61	2.45 ± 1.11	1.00 ± 0.17
		ACRB	2.45 ± 1.07	1.39 ± 0.59	3.35 ± 1.15	1.54 ± 0.37	0.68 ± 0.23
	Female	Ross	1.79 ± 0.84	1.23 ± 0.58	0.90 ± 0.25	1.30 ± 0.47	1.29 ± 0.20
		ACRB	0.84 ± 0.40	1.96 ± 0.34	2.36 ± 0.69	1.15 ± 0.42	0.44 ± 0.14
*Pit-1γ*	Male	Ross	0.87 ± 0.56	2.14 ± 1.96	3.19 ± 1.88	1.20 ± 0.84	1.00 ± 0.99
		ACRB	1.46 ± 0.86	2.18 ± 1.42	0.36 ± 0.18	1.96 ± 1.15	1.05 ± 0.63
	Female	Ross	0.74 ± 0.73	0.69 ± 0.56	0.93 ± 0.74	1.94 ± 1.11	1.71 ± 0.99
		ACRB	0.82 ± 0.82	2.72 ± 1.76	3.06 ± 1.83	2.52 ± 1.50	0.73 ± 0.44
*Total Pit-1*	Male	Ross	1.22 ± 0.67	2.56 ± 1.92	2.92 ± 1.52	1.83 ± 0.77	1.00 ± 0.63
		ACRB	1.64 ± 1.02	1.85 ± 0.95	2.78 ± 1.62	1.57 ± 0.70	0.92 ± 0.36
	Female	Ross	1.16 ± 0.76	1.45 ± 0.73	2.99 ± 1.93	1.78 ± 0.75	0.98 ± 0.37
		ACRB	0.45 ± 0.25	2.17 ± 1.27	2.54 ± 1.13	1.23 ± 0.51	1.14 ± 0.59

### Adrenocorticotropic axis

**Fig. 1:**
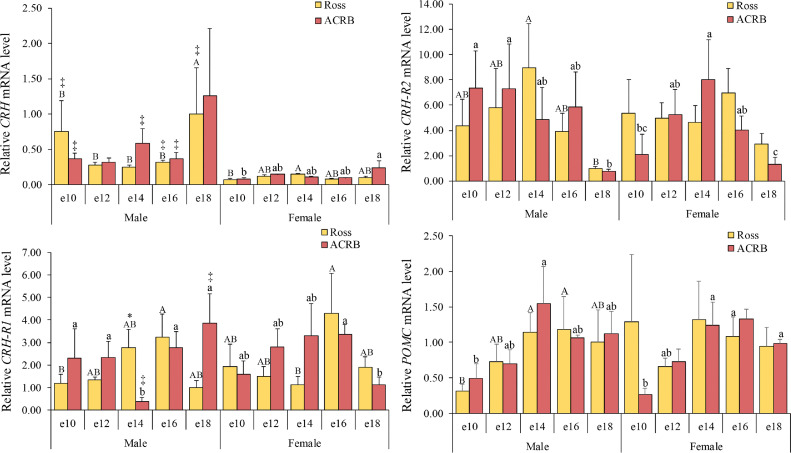
Relative mRNA levels in the adrenocorticotropic axis of hypothalamic corticotropin-releasing hormone (**CRH**), pituitary *CRH* receptors 1 and 2 (**CRH-R1** and **CRH-R2**), and pituitary pro-opiomelanocortin (**POMC**) during embryonic development. Levels of target mRNA were normalized to levels of *GAPDH* mRNA and *PGK1* mRNA for the hypothalamus and pituitary, respectively. Results are presented as the means and standard errors of the means after normalization to the mean for male e10 Ross. *P*-values of the effects on candidate gene mRNA levels are provided in Table 1. There were significant effects of age and gender on hypothalamic *CRH* mRNA levels (*P* < 0.05), while pituitary *CRH-R2* and *POMC* mRNA levels were influenced only by age (*P* < 0.05). Additionally, pituitary *CRH-R1* mRNA levels were influenced by interactions among breed, age, and gender (*P* < 0.05). Statistical significance of differences between chicken breeds at a specific age is indicated by asterisks (*), and differences between genders are indicated by double daggers (‡). Statistical significance among different ages within a breed is indicated by letters (lowercase for ACRB; uppercase for Ross).

Corticotropin-releasing hormone (**CRH**), its receptors (CRH-R1 and CRH-R2), and POMC are critical regulators of growth and body composition, playing key roles in the stress response, nutrient metabolism and partitioning, and regulation of corticosterone production (Kadhim et al., 2021). We assessed the ontogeny of hypothalamic *CRH* mRNA levels and the mRNA levels of pituitary *CRH-R1, CRH-R2*, and *POMC* during embryonic development. The relative mRNA level results for the adrenocorticotropic axis are presented in Fig. 1, and the interactions and main effects across breed, age, and gender are shown in Table 1. Hypothalamic *CRH* mRNA levels were affected by age (*P* = 0.0190) and gender (*P* < 0.0001). Overall, Ross and ACRB showed a similar pattern, with high levels at e18 and being higher in males than in females (*P* < 0.05). Pituitary *CRH-R1* mRNA levels were affected by the three-way interaction of breed, age, and gender (*P* = 0.0010). The levels were lower in Ross than in ACRB, except at e14 in males (*P* < 0.05). Age significantly influenced pituitary *CRH-R2* (*P* = 0.0045) and *POMC* (*P* = 0.0001) mRNA levels. Pituitary *CRH-R2* mRNA reached its lowest levels at the end of embryonic development (e18), whereas pituitary *POMC* increased at e12 and remained high before hatch (*P* < 0.05).

### Thyrotropic axis

**Fig. 2:**
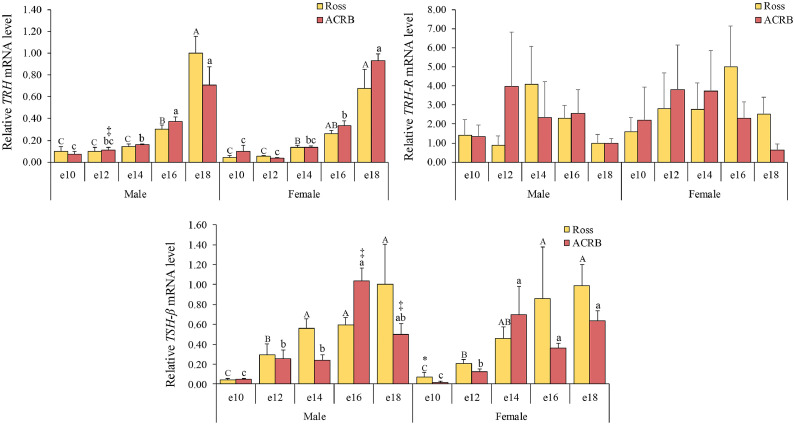
Relative mRNA levels in the thyrotropic axis of hypothalamic thyrotropin-releasing hormone (**TRH**), pituitary TRH receptor (**TRH-R**), and pituitary thyroid-stimulating hormone β-subunit (**TSH-β**) during embryonic development. Levels of mRNA were normalized to levels of *GAPDH* and *PGK1* for the hypothalamus and pituitary, respectively. Results are presented as the means and standard errors of the means after normalization to the mean for male e10 Ross. *P*-values of the effects on target gene mRNA levels are provided in Table 1. There was a significant effect of age on hypothalamic *TRH* mRNA levels (*P* < 0.05), while pituitary *TSH-β* mRNA levels were influenced by interactions among breed, age, and gender (*P* < 0.05). Statistical significance of differences between chicken breeds at a specific age is indicated by asterisks (*), and differences between genders are indicated by double daggers (‡). Statistical significance among different ages within a breed is indicated by letters (lowercase for ACRB; uppercase for Ross).

The synthesis and release of TSH from pituitary thyrotrophs in birds are intricately regulated by thyrotropin-releasing hormone (**TRH**), CRH, and SST, each acting through their respective pituitary receptors—TRH-R, CRH-R2, and SSTR2 (De Groef et al., 2003a,b). We found that hypothalamic *TRH* mRNA levels were influenced by age (*P* < 0.0001), showing a gradual increase and reaching their highest levels at e18 (*P* < 0.05). Pituitary *TSH-β* mRNA levels were significantly affected by the interactive effects of breed, age, and gender (*P* = 0.0244, Table 1). The relative mRNA level results for the thyrotropic axis are presented in Fig. 2. Overall, pituitary *TSH-β* mRNA levels were higher in Ross than in ACRB, with a significant difference observed at e10 in females (*P* < 0.05).

### Somatotropic axis

**Fig. 3:**
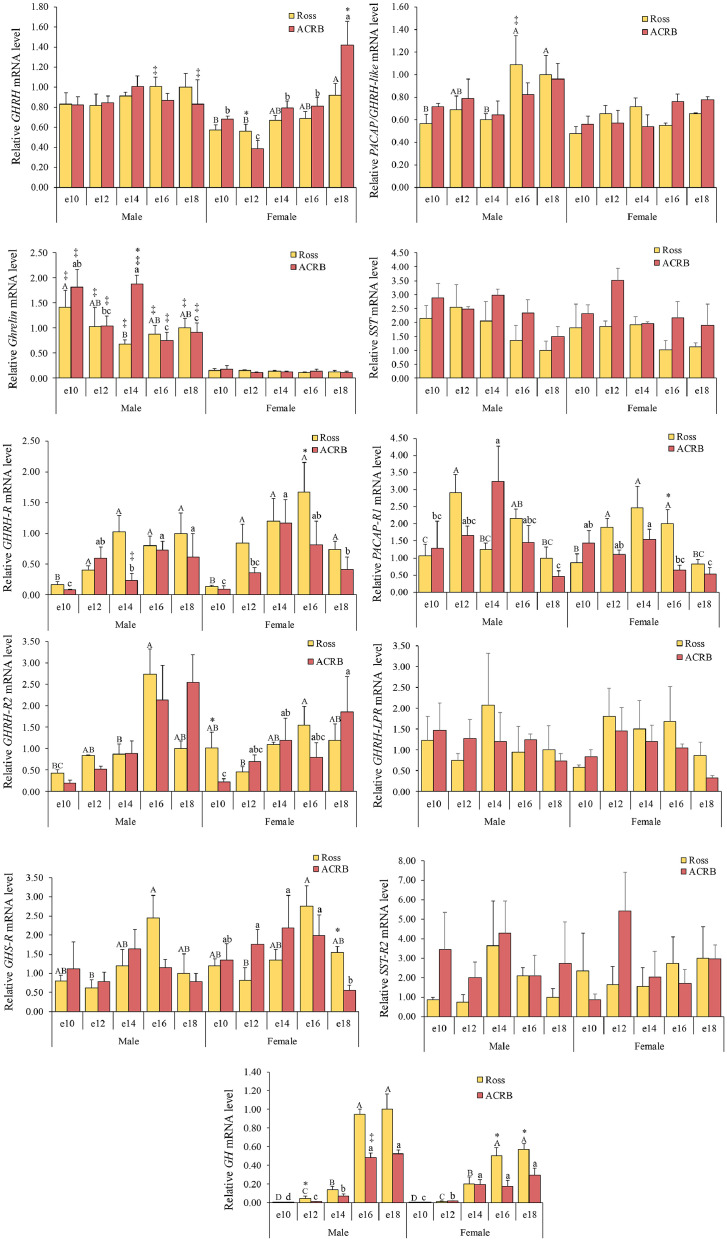
Relative mRNA levels in the somatotropic axis of hypothalamic growth hormone-releasing hormone (**GHRH**), hypothalamic pituitary adenylate cyclase-activating polypeptide/GHRH-like (**PACAP/GHRH-like**), hypothalamic ghrelin, and hypothalamic somatostatin (**SST**), pituitary *GHRH* receptor (**GHRH-R**) and GHRH receptor 2 (**GHRH-R2**), pituitary PACAP receptor 1 (**PACAP-R1**), pituitary GHRH-like peptide receptor (**GHRH-LPR**), pituitary growth hormone secretagogue receptor (**GHS-R**), pituitary SST receptor type 2 (**SSTR2**), and pituitary growth hormone (**GH**) during embryonic development. Levels of mRNA were normalized to levels of *GAPDH* and *PGK1* for the hypothalamus and pituitary, respectively. Results are presented as the means and standard errors of the means after normalization to the mean for male e10 Ross. *P*-values of the effects on target gene mRNA levels are provided in Table 1. Pituitary *GH* mRNA levels were affected by breed (*P* < 0.05). Hypothalamic *GHRH* and pituitary *GH* mRNA levels were influenced by the interaction effect of age and gender (*P* < 0.05), whereas hypothalamic *SST* and *ghrelin* mRNA levels were affected by the main effects of breed, age, and gender, respectively. Pituitary *GHRH-R2* and *PACAP-R1* mRNA levels were affected by interactions between breed and age (*P* < 0.05). Pituitary *GHS-R* and *PACAP/GHRH-like* mRNA levels were influenced by age and gender, while pituitary *GHRH-R* mRNA levels were affected by breed and age (*P* < 0.05)*.* Statistical significance of differences between chicken breeds at a specific age is indicated by asterisks (*), and differences between genders are indicated by double daggers (‡). Statistical significance among different ages within a breed is indicated by letters (lowercase for ACRB; uppercase for Ross).

GH production and release from pituitary somatotrophs are regulated by both stimulatory and inhibitory hypothalamic factors (Ranke and Wit, 2018). As in mammals, the primary stimulatory factor is GHRH, while SST serves as the main inhibitory factor (Pech-Pool et al., 2020). Additionally, GH release in chickens is stimulated by TRH and, to a lesser extent, by pituitary adenylate cyclase-activating polypeptide/GHRH-like (**PACAP/GHRH-like**) and ghrelin (Urban-Sosa et al., 2024). The relative mRNA levels of the somatotropic axis genes are shown in Fig. 3, and the interactions and main effects across breed, age, and gender are shown in Table 1. Hypothalamic *GHRH* mRNA levels were affected by the interaction between age and gender (*P* = 0.0009). Levels did not change in male embryos of either genetic line, whereas in females, they showed an increase before hatching in both lines (*P* < 0.05). Hypothalamic *PACAP/GHRH-like* mRNA levels were significantly affected by age (*P* = 0.0138) and gender (*P* = 0.0136). Levels remained stable in females, whereas in Ross males, they increased significantly between e16 and e18 (*P* < 0.05). Hypothalamic *ghrelin* mRNA levels were influenced by gender (*P* **<** 0.0001), with higher levels in males than females (*P < 0.05*). Hypothalamic *SST* mRNA levels were affected by breed (*P* = 0.0197), with higher levels in ACRB than in Ross. Pituitary *GHRH-R* mRNA levels were affected by breed (*P* = 0.0019) and age (*P* < 0.0001)*.* Levels were higher in Ross than in ACRB, with a significant increase observed between e12 and e16 *(P* < 0.05). Pituitary *GHRH-R2* (*P* = 0.0471) and *PACAP-R1* (*P* = 0.0429) mRNA levels were influenced by interactions between breed and age. Overall*,* mRNA levels of pituitary *GHRH-R2* and *PACAP-R1* were higher in Ross than in ACRB, with a significantly higher level at e10 for pituitary *GHRH-R2* and at e16 for pituitary *PACAP-R1* (*P < 0.05*). Pituitary *GHS-R* mRNA levels were significantly affected by age (*P* = 0.0112) and gender (*P* = 0.0329). Levels peaked at e16 but dropped at e18 (*P* < 0.05). Pituitary *GH* mRNA levels were affected by breed (*P* = 0.0022), with overall higher levels in Ross than in ACRB. The interaction between age and gender significantly affected pituitary *GH* mRNA levels (*P* = 0.0299), with expression increasing toward the end of embryogenesis (e16–e18). Female Ross embryos showed significantly higher levels than ACRB between e16 and e18 (*P* < 0.05), whereas no statistically significant difference was observed in males.

### Lactotropic axis

**Fig. 4:**
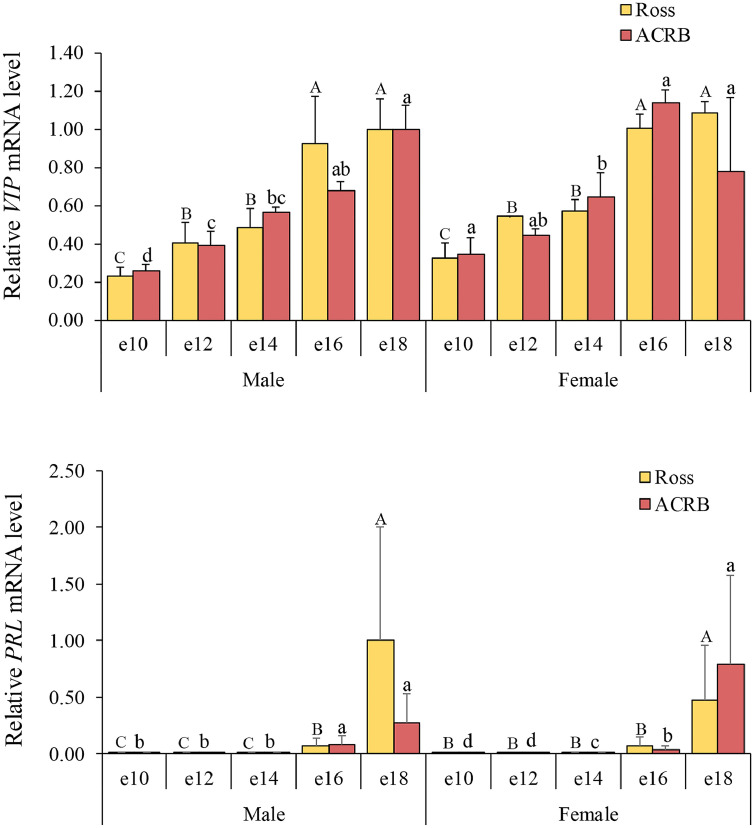
Relative mRNA levels in the lactotropic axis of hypothalamic vasoactive intestinal peptide (**VIP***)* and pituitary prolactin (**PRL**) mRNA levels. Levels of mRNA were normalized to levels of *GAPDH* and *PGK1* for the hypothalamus and pituitary, respectively. Results are presented as the means and standard errors of the means after normalization to the mean for male e10 Ross. *P*-values of the effects on target gene mRNA levels are provided in Table 1. There were significant effects of age and gender on the mRNA levels of hypothalamic *VIP* (*P* < 0.05). However, pituitary *PRL* mRNA levels were influenced only by gender (*P* < 0.05). Statistical significance among different ages within a breed is indicated by letters (lowercase for ACRB; uppercase for Ross).

VIP is recognized as the primary PRL-releasing factor in avian species (Maney et al., 1999). The relative mRNA levels for the lactotropic axis are shown in Fig. 4, and the interactions and main effects across breed, age, and gender are shown in Table 1. In this study, no significant breed effects were observed for hypothalamic *VIP* or pituitary *PRL* mRNA levels (*P* > 0.05). Hypothalamic *VIP* mRNA levels were significantly affected by both age (*P* < 0.0001) and gender (*P* = 0.0028). The activation of *VIP* mRNA expression occurred at e12 in most treatment groups, except in ACRB females, where activation began later at e14 (*P* < 0.05). Pituitary *PRL* mRNA levels were significantly influenced by age (*P* < 0.0001), showing a marked increase at the end of embryogenesis (e16–e18, *P* < 0.05).

### Gonadotropic axis

**Fig. 5:**
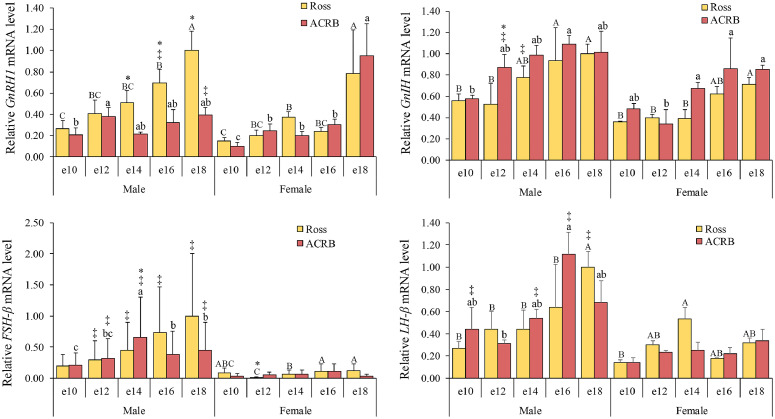
Relative mRNA levels in the gonadotropic axis of hypothalamic gonadotropin-releasing hormone (**GnRH1**) and gonadotropin-inhibiting hormone (**GnIH**) and pituitary levels of follicle-stimulating hormone β-subunit (**FSH-β**) and luteinizing hormone β-subunit (**LH-β**) during embryonic development. Levels of mRNA were normalized to levels of *GAPDH* and *PGK1* for the hypothalamus and pituitary, respectively. Results are presented as the means and standard errors of the means after normalization to the mean for male e10 Ross. *P*-values of the effects on target gene mRNA levels are provided in Table 1. There were significant effects of breed, age, and gender on the expression levels of hypothalamic *GnRH1* and *GnIH* (*P* < 0.05). Additionally, pituitary *FSH-β* mRNA levels were affected by the interaction between age and gender (*P* < 0.05), while pituitary *LH-β* mRNA levels were affected by the main effect of age and gender (*P* < 0.05). Statistical significance of differences between chicken breeds at a specific age is indicated by asterisks (*), and differences between genders are indicated by double daggers (‡). Statistical significance among different ages within a breed is indicated by letters (lowercase for ACRB; uppercase for Ross).

The gonadotropins FSH and LH are positively regulated by GnRH and negatively regulated by gonadotropin-inhibitory hormone (**GnIH**). These hypothalamic factors exert their effects through the GnRH-R and the GnIH receptors (GnIH-R1 and GnIH-R2) on pituitary gonadotrophs, thereby regulating the synthesis and release of LH and FSH (Bédécarrats et al., 2009)*.* The mRNA expression levels for the gonadotropic axis are shown in Fig. 5, and the interactions and main effects across breed, age, and gender are shown in Table 1. Hypothalamic *GnRH1* mRNA levels were significantly affected by breed (*P* = 0.0086), age (*P* < 0.0001**)**, and gender (*P* = 0.0094**)**. In males, hypothalamic *GnRH1* mRNA levels in Ross increased at e16 and peaked again at e18, whereas they increased earlier in ACRB at e12 and remained elevated until hatch. Ross males exhibited higher hypothalamic *GnRH1* mRNA levels than ACRB from e14 to e18 (*P* < 0.05). In females, hypothalamic *GnRH1* mRNA levels in Ross increased at e14, while in ACRB they rose earlier at e12 (*P* < 0.05). The hypothalamic *GnRH1* mRNA levels in both lines peaked at e18 (*P* < 0.05), but no significant differences were observed between breeds (*P* > 0.05). When comparing between sexes, hypothalamic *GnRH1* mRNA levels were higher in Ross males at e16 but lower in ACRB males at e18 (*P* < 0.05). Like hypothalamic *GnRH1*, hypothalamic *GnIH* mRNA levels also had the same effects on breed (*P* = 0.0187**)**, age (*P* < 0.0001), and gender (*P* = 0.0001). In males, levels were elevated at e16, and Ross showed lower levels than ACRB at e12 (*P* > 0.05). In females, levels increased at e14 in ACRB and e18 in Ross. When comparing between sexes, hypothalamic *GnIH* mRNA levels were higher in ACRB males at e12 (*P* < 0.05). Pituitary *FSH-β* mRNA levels were affected by interactions between breed and age (*P* < 0.0225). Ross exhibited higher levels of pituitary *FSH-β* than ACRB, except on e14 in males and e12 in females (*P* < 0.05). Pituitary *LH-β* mRNA levels were affected by age (*P* = 0.0056**)** and gender (*P* **<** 0.0001)*.* The mRNA levels of pituitary *LH-β* increased at e18 in male Ross and e16 ACRB (*P* < 0.05). In females, pituitary *LH-β* mRNA levels in Ross peaked at e14, while levels in ACRB were unchanged (*P* > 0.05). The levels of pituitary *LH-β* mRNA were higher in males than in females at e18 in Ross and e10, e14, and e16 in ACRB (*P* < 0.05).

### Appetite *and metabolism*

**Fig. 6:**
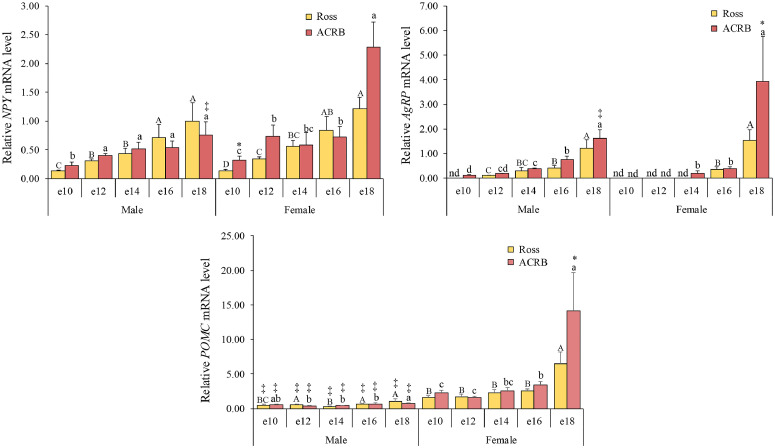
Relative mRNA levels in the hypothalamic appetite and metabolism regulating neuropeptides neuropeptide (**NPY**), agouti-related peptide (**AgRP**), and pro-opiomelanocortin (**POMC**) during embryonic development. Levels of target mRNA were normalized to levels of *GAPDH* mRNA. Results are presented as the means and standard errors of the means after normalization to the mean for male e10 Ross. *P*-values of the effects on target gene mRNA levels are provided in Table 1. They were affected by the main effect of breed, age, or gender on the expression levels of hypothalamic *NPY* and *AgRP* (*P* < 0.05), while hypothalamic *POMC* mRNA levels were affected by the interaction effect of breed and gender (*P* < 0.05). Statistical significance of differences between chicken breeds at a specific age is indicated by asterisks (*), and differences between genders are indicated by double daggers (‡). Statistical significance among different ages within a breed is indicated by letters (lowercase for ACRB; uppercase for Ross). Undetectable mRNA expression is indicated by not detectable (nd).

Orexigenic AgRP/NPY neurons stimulate food intake, whereas anorexigenic POMC neurons suppress feeding (Boswell et al., 2002; Richards, 2003). Relative mRNA levels for the appetite and metabolism genes are shown in Fig. 6, and the interactions and main effects across breed, age, and gender are shown in Table 1. Hypothalamic *NPY* mRNA levels were significantly affected by breed (*P* = 0.0441), age (*P* < 0.0001**)**, and gender (*P* = 0.0033**)**. Overall, hypothalamic *NPY* mRNA levels were higher in ACRB than in Ross, with ACRB females showing higher levels than males at e18 (*P* < 0.05). Hypothalamic *AgRP* mRNA levels were affected by breed (*P* = 0.0160**)** and age (*P < 0.0001*). In Ross, hypothalamic *AgRP* mRNA levels initiated at e12 in males and at e16 in females. In ACRB, hypothalamic *AgRP* mRNA levels initiated at e12 in males, whereas they initiated at e16 in females. Overall, hypothalamic *AgRP* mRNA levels were higher in ACRB than in Ross (*P* < 0.05). When comparing between sexes, hypothalamic *AgRP* mRNA levels were higher in ACRB females than in males at e18 (*P* < 0.05). The interaction between breed and gender was significant for hypothalamic *POMC* mRNA levels (*P* = 0.0350). Across embryonic development in both breeds, females exhibited higher hypothalamic *POMC* mRNA levels than males (*P* < 0.05). Additionally, at e18, female ACRB showed higher *POMC* mRNA levels than female Ross birds (*P* < 0.05).

## Discussion

Selective breeding of broilers has enhanced productivity but has also influenced the neuroendocrine system. A deeper understanding of how genetic selection affects neuroendocrine function could enable the development of breeding strategies that further improve production efficiency in broilers. To achieve this, the ontogeny of gene expression in the hypothalamus and anterior pituitary of a modern commercial broiler line (Ross 708) and the oldest established control strain for meat-type chickens, ACRB birds, was investigated. We report that expression of genes involved in four of the hypothalamic-pituitary trophic axes – the adrenocorticotropic axis, the thyrotropic axis, the somatotropic axis, and the gonadotropic axis – as well as appetite and metabolism control, differed between Ross broilers and ACRB birds during embryonic development. Each of these plays a role in regulating growth, body composition, and metabolism in chickens.

The neuroendocrine adrenocorticotropic axis, comprising hypothalamic CRH and pituitary ACTH and their downstream receptors and targets, plays a pivotal role in regulating the stress response, nutrient metabolism and partitioning, and body growth and composition (Kadhim et al., 2019; Grammatopoulos, 2012). Importantly, adrenal glucocorticoids under control of this axis mobilize lipids and proteins for gluconeogenesis by the liver. A previous study reported that CRH-producing neurons and corticotrophs, along with TRH-producing neurons and thyrotrophs, are activated to generate the energy needed to maintain homeostasis in birds experiencing stress (Kadhim and Kuenzel, 2022). The hypothalamic-pituitary-adrenal (**HPA**) axis is functionally active prior to hatching in chicks. The pituitary gland becomes increasingly important in regulating both basal hormone levels and the stress response, while the hypothalamus is essential for mediating the hormonal response to stress (Wise and Frye, 1973). Consistent with these early findings, the mRNA levels of hypothalamic *CRH*, pituitary *CRH-R2*, and *POMC* in the current study were found to be age-dependent. High levels of hypothalamic *CRH* and pituitary *POMC* mRNA before hatching likely prepare the embryo for the metabolic and physiological demands of post-hatch life for supporting the rapid growth and energy requirements needed for survival immediately after hatch (Jenkins and Porter, 2004). Notably, the higher hypothalamic *CRH* mRNA expression observed in males compared to females may reflect differences in energy requirements between the sexes (Shalev and Pasternak, 1998). On the other hand, pituitary *CRH-R2* is expressed in thyrotrophs and mediates CRH-stimulated TSH release (De Groef et al., 2003a,b). The decrease in pituitary *CRH-R2* mRNA observed before hatch may reflect its role in regulating CRH-induced TSH release from pituitary thyrotrophs, as described by Ellestad et al. (2011). In the neuroendocrine adrenocorticotropic axis, hypothalamic CRH stimulates pituitary corticotrophs to produce ACTH via CRH-R1 (De Groef et al., 2003a). Lower pituitary *CRH-R1* mRNA levels in embryonic modern broilers may reduce corticosterone, leading to lower heat production, reduced stress responses, and improved feed efficiency, supporting faster growth (Khan et al., 2015; Vaccaro et al., 2022b). Male Ross embryos have heavier embryonic weight than ACRB embryos from e14 to e18 (Vaccaro et al., 2022b). The elevated pituitary *CRH-R1* expression at e14 in males may reflect the effects of genetic selection, providing enhanced metabolic support during a critical growth phase before subsequent downregulation helps reduce heat production and stress sensitivity later in development in male Ross. These findings suggest an evolving role of the HPA axis in metabolic regulation during embryonic development.

The thyrotropic axis regulates metabolic rate and plays a major role in regulating growth in chickens (Scanes et al., 2021). The regulation of TSH in birds is influenced by several hypothalamic factors, including TRH, CRH, and SST (De Groef et al., 2003a,b). Hypothalamic TRH and CRH play a crucial role in regulating pituitary *TSH-β* mRNA expression and secretion during late embryonic development, and the progressive increase in TRH during this period supports TSH production to enhance circulating thyroid hormone levels (Grommen et al., 2008; Ellestad et al., 2011). The gradual increase of hypothalamic *TRH* mRNA levels during embryonic development in this study highlights the developmental significance of the thyrotropic axis in preparing for thyroid function in response to metabolic demands (Zhao et al., 2024). Higher pituitary *TSH-β* mRNA levels in Ross than in ACRB may likely reflect genetic selection and the establishment of negative feedback mechanisms due to increased thyroid hormone levels during embryonic development (Grommen et al., 2008; Ellestad et al., 2011). In general, our findings indicate that the embryonic ontogeny and function of the thyrotropic axis have been advanced through the genetic selection of modern broilers.

The somatotropic axis plays a major role in the regulation of growth in chickens. For example, dwarf chickens result from a mutation in the GH receptor (Burnside et al., 1991). In pituitary somatotrophs, GH secretion is regulated by a complex neuroendocrine system, primarily through the opposing actions of GHRH and SST, which exert stimulatory and inhibitory effects, respectively (Müller et al., 1999). Hypothalamic *GHRH, PACAP/GHRH-like*, and *ghrelin* are well known to stimulate the production and release of GH from the pituitary (Steyn et al., 2016). However, mRNA levels of hypothalamic *GHRH, PACAP/GHRH-like, ghrelin*, and pituitary *GHS-R* were not influenced by breed in this study. The different expression patterns of these genes for the somatotropic axis mutation may influence GH secretion and potentially contribute to different growth rates between male and female chickens after hatch (Reiprich, 1995). Interestingly, the mRNA level of SST, an inhibitor of GH production, was lower in Ross than ACRB embryos of both sexes, accompanied by increased expression of pituitary *GHRH-R, GHRH-R2*, and *PACAP-R1*. In addition, overall pituitary *GH* mRNA levels were observed to be higher in Ross than in ACRB at the end of embryogenesis. Taken together, genetic selection may have reduced the inhibitory effect of hypothalamic SST on GH secretion and enhanced the activation of hypothalamic GH-releasing factor receptors in the pituitary of Ross embryos during development. The effect of breed and age on mRNA levels in the somatotropic axis suggests that genetic selection has influenced the regulation of GH production and release in embryonic chickens (Jia et al., 2018). As embryonic development represents one-third of the life of a modern broiler chicken, this advancement in the somatotropic axis may play a crucial role in modulating growth during both embryonic and post-hatch development.

The gonadotropic axis regulates reproduction, but it also regulates the production of steroid hormones that affect muscle accretion. The gonadotropic axis, which involves GnRH and GnIH regulation of LH and FSH synthesis, is crucial for reproductive development and regulation of gonadal function. They are positively regulated by GnRH1 and negatively regulated by GnIH (Dunn et al., 2009). In males, the lower hypothalamic *GnRH1* mRNA levels observed in ACRB compared to Ross at e12 coincided with higher hypothalamic *GnIH* mRNA levels, suggesting that GnRH release during embryogenesis in ACRB may be suppressed by GnIH at this stage. Genetic selection in broilers may enhance hypothalamic *GnRH1* mRNA expression at the later stages of embryonic development (e14–e18) by suppressing hypothalamic GnIH release around e12, thereby increasing *FSH-β* mRNA expression. Since gonadotropins play a key role in regulating growth and body composition (Rogol et al., 2002), the mechanism may explain heavier Ross than ACRB embryos from e14 to e18 (Vaccaro et al., 2022b). This suggests that genetic selection in broilers has modified gene expression patterns in the gonadotropic axis, potentially influencing the development and performance of the reproductive system, including the ontogeny and levels of gonadal steroids, with distinct patterns observed between males and females (Zhang et al., 2018).

In contrast to the corticotropic, thyrotropic, somatotropic, and gonadotropic axes, no effect of breed or its interactions with age or gender were found in the lactotropic axis in the current study. In both mammals and birds, the lactotropic axis regulates PRL secretion through hypothalamic VIP acting on pituitary lactotrophs (Samson et al., 1989; Tong et al., 1998). In this study, we observed that pituitary *PRL* mRNA levels increased following the activation of hypothalamic *VIP* mRNA, suggesting that elevated hypothalamic *VIP* may contribute to the induction of pituitary *PRL* gene expression during late embryonic development (Ellestad et al., 2011). Since no breed effects were detected on the mRNA levels of the lactotropic axis, and this axis is initiated around e18 (Ellestad et al., 2011), these findings suggest that the activation of hypothalamic *VIP* and pituitary *PRL* expression may contribute to the development of lactotrophs during embryogenesis (Woods and Porter, 1998), rather than to embryonic growth.

Appetite-regulatory neuropeptides, including NPY, AgRP, and POMC, are expressed in the ARC nucleus in the hypothalamus and act coordinately to regulate energy balance (Schwartz, 2001). NPY and AgRP signaling promotes feed intake and suppresses adipolysis and metabolic activity; this pathway is negatively regulated by leptin and stimulated by ghrelin. Beyond appetite control, AgRP also influences the neuroendocrine axis by promoting ACTH and glucocorticoid secretion and inhibiting TRH activity, underscoring its integrative role in metabolism and hormonal regulation (Deem et al., 2022). POMC is a precursor of ACTH, β-endorphin, α-melanocyte-stimulating hormone (**α-MSH**), and β-MSH (Zhan et al., 2013). In avian species, hypothalamic POMC contributes more to ACTH and β-endorphin production than to α-MSH, and increased *POMC* expression has been associated with reduced body mass and androgenic effects (Boswell and Dunn, 2017; Piórkowska et al., 2020). In addition, it has been reported that growth rate and feed intake influence the expression of hypothalamic appetite-regulatory genes in adult broilers (Piórkowska et al., 2020). ACRB chicken has a lower growth rate and feed intake than modern broilers (Collins et al., 2014). However, the developmental profile of hypothalamic appetite regulation in chick embryos has not been reported. Here, we demonstrate for the first time that hypothalamic *NPY, AgRP*, and *POMC* are activated during embryonic development, with higher expression levels observed in ACRB compared with Ross. These findings indicate that ACRB exhibits stronger appetite- and metabolism-related signaling than Ross, which may contribute to differences in early post-hatch appetite programming between the two lines.

## Conclusions

Our findings provide valuable insights into the developmental regulation of key neuroendocrine axes that govern growth and other essential physiological processes in chickens. The results demonstrate that genetic selection of modern broilers not only influenced physical traits but also altered the neuroendocrine regulation of the adrenocorticotropic, somatotropic, thyrotropic, and gonadotropic axes, as well as appetite and metabolism control during embryonic development. These changes may impact growth, metabolism, feed efficiency, and body composition in modern broilers.

## CRediT authorship contribution statement

**Panpradub Sinpru:** Writing – original draft, Methodology, Formal analysis. **Kristen Diehl:** Writing – review & editing, Formal analysis. **Laura E. Ellestad:** Writing – review & editing, Methodology. **Tom E. Porter:** Writing – review & editing, Supervision, Project administration, Funding acquisition, Conceptualization.

## Disclosures

The authors declare the following financial interests/personal relationships which may be considered as potential competing interests: Tom E. Porter reports financial support was provided by US Department of Agriculture National Institute of Food and Agriculture (USDA-NIFA). If there are other authors, they declare that they have no known competing financial interests or personal relationships that could have appeared to influence the work reported in this paper.
